# Using computational psychiatry for identifying risk factors for depressive disorder

**DOI:** 10.1192/j.eurpsy.2025.1341

**Published:** 2025-08-26

**Authors:** I. Marinić, L. Mužinić Marinić

**Affiliations:** 1 Department of Psychiatry, Dubrava University Hospital, Zagreb, Croatia

## Abstract

**Introduction:**

Computational psychiatry uses computer models to improve the understanding, diagnosis, and treatment of mental health disorders. These models can integrate large datasets from various sources, including genetic, neurobiological, and environmental factors, to predict the likelihood of developing depression.

**Objectives:**

The aim of the study is to explore the potential of computational psychiatry methods in identifying risk factors for the development of depressive disorders.

**Methods:**

A review of relevant studies was conducted using the PubMed database. The search focused on articles examining computational psychiatry approaches, particularly those assessing risk factors associated with the onset of depressive disorders.

**Results:**

The study highlights computational models that show potential in identifying risk factors for depressive disorders.

**Conclusions:**

Computational psychiatry offers new insights into identifying risk factors for psychiatric disorders and has the potential to contribute to the prevention and treatment of depressive disorders. However, further research is needed to improve the generalizability and applicability of the models.

**Disclosure of Interest:**

None Declared

